# Detecting imipenem resistance in *Acinetobacter baumannii *by automated systems (BD Phoenix, Microscan WalkAway, Vitek 2); high error rates with Microscan WalkAway

**DOI:** 10.1186/1471-2334-9-30

**Published:** 2009-03-16

**Authors:** Canan Kulah, Elif Aktas, Fusun Comert, Nagihan Ozlu, Isin Akyar, Handan Ankarali

**Affiliations:** 1 Zonguldak Karaelmas University, Faculty of Medicine, Department of Microbiology and Clinical Microbiology, Zonguldak, Turkey; 2 Acibadem Labmed Clinical Laboratories, Istanbul, Turkey; 3 Zonguldak Karaelmas University, Faculty of Medicine, Department of Biostatistics, Zonguldak, Turkey

## Abstract

**Background:**

Increasing reports of carbapenem resistant *Acinetobacter baumannii *infections are of serious concern. Reliable susceptibility testing results remains a critical issue for the clinical outcome. Automated systems are increasingly used for species identification and susceptibility testing. This study was organized to evaluate the accuracies of three widely used automated susceptibility testing methods for testing the imipenem susceptibilities of *A. baumannii *isolates, by comparing to the validated test methods.

**Methods:**

Selected 112 clinical isolates of *A. baumanii *collected between January 2003 and May 2006 were tested to confirm imipenem susceptibility results. Strains were tested against imipenem by the reference broth microdilution (BMD), disk diffusion (DD), Etest, BD Phoenix, MicroScan WalkAway and Vitek 2 automated systems. Data were analysed by comparing the results from each test method to those produced by the reference BMD test.

**Results:**

MicroScan performed true identification of all *A. baumannii *strains while Vitek 2 unidentified one strain, Phoenix unidentified two strains and misidentified two strains. Eighty seven of the strains (78%) were resistant to imipenem by BMD. Etest, Vitek 2 and BD Phoenix produced acceptable error rates when tested against imipenem. Etest showed the best performance with only two minor errors (1.8%). Vitek 2 produced eight minor errors(7.2%). BD Phoenix produced three major errors (2.8%). DD produced two very major errors (1.8%) (slightly higher (0.3%) than the acceptable limit) and three major errors (2.7%). MicroScan showed the worst performance in susceptibility testing with unacceptable error rates; 28 very major (25%) and 50 minor errors (44.6%).

**Conclusion:**

Reporting errors for *A. baumannii *against imipenem do exist in susceptibility testing systems. We suggest clinical laboratories using MicroScan system for routine use should consider using a second, independent antimicrobial susceptibility testing method to validate imipenem susceptibility. Etest, whereever available, may be used as an easy method to confirm imipenem susceptibility.

## Background

*Acinetobacter baumannii *infections mostly affect debilitated patients in intensive care units and are associated with high mortality rates [1,2]. *A. baumannii is *difficult to control and treat because of its prolonged environmental survival and its ability to develop resistance to multiple antimicrobial agents [3]. Carbapenems are considered the gold standard treatment for multidrug resistant (MDR) *A. baumannii *infections, imipenem being the most active agent [1,3]. However, reports of imipenem-resistant *A. baumannii *strains have been increasing during the past few years, and these isolates are often multidrug resistant MDR [3-7]. Inappropriate antimicrobial treatment increases mortality in patients with bacteraemia [8]. Reliable susceptibility testing results remains a critical issue for the clinical outcome for these patients.

Automated systems have been increasingly used in many clinical laboratories for species identification of the bacteria and susceptibility testing. These systems are able to decrease the labour and in-laboratory time compared to that required for standardized methods and help the physicians guide efficient antimicrobial therapy based on rapid and convenient results [9]. On the other hand, probable errors reported by test system can have serious implications for the clinical outcome for patients.

Currently MDR *Acinetobacter*s are among the most frequent nosocomial pathogens in our institution. After the introduction of the MicroScan WalkAway system for susceptibility testing in 2006, we noted a considerable proportion of discordant results in imipenem susceptibilities of *Acinetobacter*s compared to disk diffusion results. Upon this observation, this study was organized to evaluate the accuracies of three widely used automated susceptibility testing methods for testing the imipenem susceptibilities of *Acinetobacter baumannii *isolates, by comparing to the validated test methods.

## Methods

One hundred twelve non dublicate clinical isolates of *A. baumanii *collected between January 2003 and May 2006 were tested to confirm imipenem susceptibility results. These isolates were selected retrospectively among the strain collection derived from the clinical samples submitted to the microbiology laboratory for routine diagnostic procedures. *A. baumannii *isolates were previously characterized molecularly by PFGE method, and the selected strains were predominantly representing different *A. baumannii *genotypes found in this institution including both epidemic and sporadic clones. This research has been performed with the approval of Zonguldak Karaelmas University, Application and Research Hospital Ethics Committee. Identification of *A. baumannii *was performed according to conventional microbiological methods and confirmed by API 20 NE (bioMerieux Inc, France) [10]. Strains were tested against imipenem by broth dilution, disk diffusion, Etest (AB Biodisk, Solna, Sweden) and by the automated systems BD Phoenix (Becton Dickinson Diagnostic Systems, Sparks, MD, USA), MicroScan WalkAway (Dade Behring INC. West Sacramento, CA, USA) and Vitek 2 (bioMe'rieux, Marcy l'Étoile, France). The reference broth microdilution (BMD) test was performed using in-house prepared panels according to CLSI [11,12]. Imipenem was kindly provided by the manufacturer (Merck Sharp & Dohme, Madrid, Spain). Ouality control strains included *P. aeruginosa *ATCC 27853 and *E. coli *ATCC 25922 as recommended by CLSI [11]. Susceptibility of the isolates to the following antibacterial agents was tested by disk-diffusion (DD) method using discs (Oxoid, UK), and interpreted as recommended by CLSI [11]; imipenem (IPM, 10 μg), meropenem (MEM, 10 μg), ceftazidime (CAZ, 30 μg), cefepime (FEP, 30 μg), piperacillin (PRL, 100 μg), cefotaxime (CTX, 30 μg), ciprofloxacin (CIP, 5 μg), levofloxacin (LEV, 5 μg), gentamicin (CN, 10 μg), tetracyclin (TE, 30 μg), trimethoprim-sulfamethoxazole (SXT, 1.25/23.75 μg). The study was performed in three laboratories; for MicroScan WalkAway in Zonguldak Karaelmas University Hospital, Zonguldak, for BD Phoenix in Acibadem Labmed Clinical Laboratory, Istanbul, and for Vitek2 in bioMe'rieux Laboratory, Istanbul. The organisms were tested in a blinded fashion in each laboratory according to the procedures recommended by the manufacturers. All systems were tested with inocula from the same subculture. In addition to the WalkAway automated reading, manual readings were performed on all MicroScan panels and each isolate was tested at least twice, at different times.

Data were analysed by comparing the results from each test method to those produced by BMD test.

SPSS (ver. 11.5) programme was used for all calculations. Kappa tests was used for agreement. If P values less than 0.05 it was accepted statisticaly significant.

Very major errors were considered when an organism was defined as resistant by the reference method but was categorized as susceptible with the tested system. Major errors were defined when an organism found to be susceptible by the reference method was considered resistant with the system. Minor errors occurred when an organism was considered susceptible or resistant either by the reference microdilution method or with the tested system but intermediate by the other method. An overall category error rate of < 10% was considered for an acceptable performance of susceptibility tests, including ≤1.5% of very major errors and ≤3.0% major errors [13].

## Results

MicroScan performed correct identification of all (n = 112) *A. baumannii *strains while Vitek 2 unidentified one strain, and Phoenix unidentified two strains and misidentified two strains.

The BMD testing showed that 25 (22%) strains were susceptible to imipenem while 87 (78%) were resistant. MIC_50 _and MIC_90 _for imipenem was 32 μg/ml and 64 μg/ml respectively. Compared to the BMD results all other test systems in the study produced errors when *A. baumannii *was tested against imipenem. Etest showed the best performance with only two minor error (1.8%). Vitek2 produced eight minor errors(7.2%). BD Phoenix produced three major errors (2.8%). DD produced two very major errors (1.8%) and three major errors (2.7%). MicroScan showed the worst performance in susceptibility testing with 28 very major errors (25%) and 50 minor errors (44.6%). The error rates resulted by the three commercial automated systems and two validated methods are listed in Table 1. When the results were evaluated upon the acceptable performance criteria for susceptibility tests; the error rates of BD Phoenix and Vitek2 were in acceptable limits while MicroScan performed unacceptable results with high very major and minor error rates in detecting imipenem susceptibility.

**Table 1:** Types of errors produced when testing imipenem susceptibilities of *A. baummanii *isolates by three commercial automated systems and two validated methods.

	**No (%) of indicated type of error compared to BMD result**			
		
**System/method **(no. of strains tested)	**Very major**	**Major**	**Minor**	**Kappa and P values**
	
BD Phoenix (108)	0	3 (2,8)	0	κ = 0.919 P < 0.0001
MicroScan WalkAway (112)	28 (25)^a^	0	50 (44,6)^a^	κ = 0.158 P < 0.0001
Vitek 2 (111)	0	0	8 (7,2)	κ = 0.822 P < 0.0001
				
Etest (112)	0	0	2 (1,8)	κ = 0.950 P < 0.0001
Disk diffusion (112)	2 (1,8)^a^	3 (2,7)	0	κ = 0.870 P < 0.0001

^a^Unacceptable levels of error

Susceptibility rates of the isolates against the given antibiotics; by DD method and the three automated sytems, are shown in Table 2. Variations in susceptibility rates when tested by DD and three automated sytems are also displayed in the table. Discordant susceptibility rates were observed mostly in imipenem, meropenem, cefepime and trimethoprim-sulfamethoxazole.

**Table 2:** Susceptibilities by disk diffusion and three automated sytems against the antibiotics tested

	IMP	MEM	PRL	CAZ	FEP	CTX	CIP	LEV	CN	TE	SXT
**Disk diffusion**											
Susceptible (%)	21,4	19,6	3,6	3,6	41,1	3,6	14,3	26,8	58	14,3	29,5
Intermediate (%)	-	-	-	1,8	24,1	0,9	8,9	15,2	1,8	4,5	3,6

**BD Phoenix**											
Susceptible (%)	20,4	19,4	1,8	3,7	5,6	-	3,6	25,9	50	14,8	40,7
Intermediate (%)	-	-	3,6	2,8	50	4,5	19,4	13,9	5,6	11,1	-

**MicroScan WalkAway**											
Susceptible (%)	46,4	22,3	1,8	7,1	11,6	-	4,5	26,8	56,3	18,8	33,9
Intermediate (%)	44,6	23,2	3,6	8,9	50	4,5	9,8	13,4	4,5	3,6	3,6

**Vitek 2**											
Susceptible (%)	22,5	32,4	2,7	3,6	16,2	0,9	4,5	27,9	60,4	24,3	49,5
Intermediate (%)	7,2	64,9	2,7	2,7	45	3,6	3,6	24,3	1,8	7,2	-

Numbers of discordances between the results of DD and the three automated systems in susceptibility testing to the given antibiotics are exhibited in Table 3. For trimethoprim-sulfamethoxazole susceptibilities; high numbers of major discordances were displayed between the DD and all three automated systems and also between the automated systems. Minor discordances were mostly observed for meropenem, levofloxacin, cefepime and tetracyclin.between the three automated systems.

**Table 3:** Correlation of the results of disk diffusion (DD) and automated systems in susceptibility testing to the antibiotics tested: Numbers of discordances.

	Discordances between DD and the three automated systems						Discordances between the three automated systems					
	**DD/Phoenix**		**DD/MicroScan**		**DD/Vitek2**		**Vitek2/MicroScan**		**Vitek2/Phoenix**		**Phoenix/MicroScan**	
**Antibiotics**	Major*	Minor**	Major*	Minor**	Major*	Minor**	Major*	Minor**	Major*	Minor**	Major*	Minor**
**MEM**	3	30	7	62	15	72	3	62	4	62		31
**PRL**		4		4		3		1		1		2
**CAZ**		3	3	10	1	3	2	13	1	2	1	13
**FEP**	9	41	4	43	7	34	1	16	3	16	1	20
**CTX**	1	4	1	6	1	4	1	2	1	2	1	2
**CIP**	1	17	7	9	9	14		11		19		15
**LEV**	1	25		24	1	24		21		22		17
**CN**	7	8	4	5	5	3	1	5	5	6	3	7
**TE**	5	7	8	5	11	11	3	10	2	18	4	10
**SXT**	15	70	11	4	23	60	17		13		12	

* Discordances were defined as "major" when *A. baumannii *was found to be susceptible by one method/system and resistant with the other method/system.

** Discordances were defined as "minor" when *A. baumannii *was found to be susceptible or resistant by one method/system and intermediate with the other method/system.

*** Imipenem was not included in the table; types of errors in susceptibility testing to imipenem by all systems can be derieved in detail from Table 1.

## Discussion

Treatment of severe nosocomial infections increasingly depends on carbapenems. Despite the high level resistance in *Acinetobacter spp*., carbapenems are still considered as the most active agents [14-17]. On the other hand, emergence of resistance to carbapenems is of serious concern [3]. Outbreaks of imipenem-resistant *Acinetobacter *have been reported worldwide and carbapenem resistance among *A*. *baumannii *can be endemic in certain hospitals as well [3,18]. Thus, besides the clinical outcome and cost, reliable susceptibility testing results remains a critical issue for hospital infection control and surveillance programs.

Reproducibility, the ability to track results, potential impact on the workflow and the availability of rapid results favor the use of automatic systems in the microbiology laboratory [9]. Rapid identification and susceptibility testing can have a significant impact on the management of infections, especially those caused by antibiotic-resistant bacteria. The development of expert automated systems has allowed an increase both in the reproducibility and in the reliability of the results. Unfortunately numerous studies have reported errors of various automated systems when several organism-antimicrobial combinations tested[9,19-22].

Discrepancies of resistance to imipenem by automated systems have been described in enteric species, previously [19,23]. False resistance to imipenem was documented in *Pseudomonas aeruginosa, A. baumanni, Proteus mirabilis *whereas false susceptibility was reported in *Klebsiella pneumoniae *[2,24,25]. It has been shown that false resistance was due to the imipenem degradation over time, which resulted false increases in MICs of imipenem [24]. Whereas, false susceptible results by the automated systems in *K. pneumoniae *isolates were attributed in part to low inoculum size [26,27].

Treatment of *A. baumannii *infections is guided foremost by *in vitro *antimicrobial susceptibility assays. Although the BD Phoenix, MicroScan Walkaway and Vitek are commonly used automated test systems in antimicrobial susceptibility, few studies have been reported for discrepancies of resistance to imipenem in *A. baumannii *[2,28]. It was also supposed that the prediction of resistance in *A. baumannii *based upon susceptibility assays might be less certain than for other bacteria [18].

According to our results, it can be stated that, overall, the Vitek2 and Phoenix system reliably carried out susceptibility testing for imipenem against *A. baumanni*. The MicroScan WalkAway system generally failed to accurately detect imipenem resistance among our collection of carbapenem resistant *A. baumanni *isolates. Up to our knowledge this is the first description of considerable false susceptibility results to imipenem in *A. baumannii *by an automated system.

This study puts forth discordant results between the three widely used automated susceptibility testing methods for testing the imipenem susceptibilities of *A. baumannii *isolates for consideration. According to the fact that the study was performed in three laboratories; standardization difficulties might be questioned. However, all systems were tested with inocula from the same subculture and procedures were performed stringly according to the manufacturers recommendations for both inoculum preparation and technical details at every laboratory. All strains for which the commercial MIC results were discrepant with MIC results from the BMD reference method were retested by using all methods. Besides, the BMD, Etest, DD methods were performed at the same laboratory with the automated system which displayed unacceptable error rates.

Although our results clearly displayed that the MicroScan system failed to accurately detect imipenem resistance among our *A. baumanni *isolates; it was not possible to attribute the invalid MIC results to a certain factor in our case. Previously it was suggested that the variability in detecting imipenem resistance by automated systems was partly a result of underinoculating the panels and was emphasised that appropriate inoculum size was a critical factor for achieving accurate results especially when cell wall-active antimicrobials were tested [19,26]. False-susceptible results based on these factors were noted with both VITEK and MicroScan WalkAway systems [26,27]. Based on this data; our initial effort was careful attention to inoculum. Prompt Inoculation System-D (3 M Company, St. Paul, MN) is generally used practically for inoculum preparation in MicroScan systems. We repeated imipenem susceptibility testing of all discordant isolates in our study using the MicroScan System, with inocula prepared using both Prompt Inoculation System-D and the CLSI recommended protocol. However, repeat testing of isolates with careful attention to inoculum appeared not to improve results. On the other hand, the same bacterial inoculum prepared per each isolate according to CLSI was used concurrently for the Etest and BMD and also retested by the MicroScan WalkAway system, which gave high error rates. According to our results, it should be stated that inoculum size, indeed, cannot be attributed to the inaccurate results in our case.

As recommended by the manufacturer, manual readings were performed on all MicroScan panels as well. It was previously noted that MicroScan yielded several very major errors by the automated instrument readings but only minor or no errors when the tests were read visually[23]. The probable reason for manuel reading was based on the instrument's threshold for identifying bacterial growth [23]. At the same time growth control wells were also checked on each panel. Technical maintenance was carried out by the manufacturer's service and a selected group of the discordant *A. baumanni *strains was also tested by the application specialist. Nevertheless these efforts appeared not to be able to improve the results performed by MicroScan WalkAway system. Yet we remain unable to explain the high rate of very major and minor errors by MicroScan system Speculating other probable reasons; high false sensitivity of *A. baumannii *to imipenem might be due to a higher-than-expected or inconsistent concentration of imipenem used in the system, which remains to be further illuminated by the manufacturer. Improper plates and other probable technical errors are other issues needs to be addressed. However it could be noted that our results may only apply under the circumstances in our laboratory as well as the shipping conditions during the study.

According to our results; DD method produced an unacceptable rate of very major errors (1.8%), slightly higher than the acceptable limit (1.5%), that can be reconsidered accounting the total number of the isolates. In consideration of low cost and requirement of no special equipment; DD method, available in most laboratories, seems to be a useful method for susceptibility testing of *A. baumannii *to imipenem. However, our data suggest that traditional disk diffusion method may result in very low rates of very major errors. Etest method, whereever available, may be used as an accurate testing method to confirm questionable results generated by automated methods and DD for susceptibility testing of *A. baumannii *to imipenem.

It is noteworthy that; inconsistent results with DD and by all automated systems in susceptibilities to antibiotics other than imipenem were also observed in this study; mainly in susceptibilities to trimethoprim-sulfamethoxazole, meropenem, cefepime and levofloxacin; consecutively. Besides when compared to each other; discordances between the three automated systems were also encountered mostly with these antibiotics. Previously high minor error levels were already noted for automated systems when testing β-lactam antimicrobials in *P. aeruginosa *[19,22,23]. However, the accuracy of the automated systems in susceptibility testing of *A. baumannii *against the aforementioned antibiotics should be further evaluted with other studies by using the reference BMD method.

## Conclusion

Testing difficulties in susceptibility testing do exist in automated susceptibility testing systems. Reporting errors can have serious implications for the clinical outcome for patients. We suggest clinical laboratories using MicroScan automated system for routine use should consider using a second, independent antimicrobial susceptibility testing method to validate imipenem susceptibility. Disk diffusion method may result in very low rates of very major errors. Etest as an easy method may be used to confirm imipenem susceptibility. If treatment failure with carbapenems is observed for isolates of *A. baumanni *that were previously reported as susceptible to carbapenems, repeat testing with a nonautomated method should be warranted.

## Competing interests

The authors declare that they have no competing interests.

## Authors' contributions

CK conceived the study, and participated in its design, coordination, carrying out and drafted the manuscript. EA carried out MIC testing and participated in the design of the study. FB participated in coordination and the MIC testing. NO carried out the automated tests and participated in the design. IA carried out BD Phoenix test. HA participated in the design of the study and performed the statistical analysis. All authors read and approved the final manuscript.

## About the authors

Canan Kulah: Ass. Asc. Prof. Dr. of Medical Microbiology

Elif Aktas: Ass. Asc. Prof. Dr. of Medical Microbiology

Fusun Comert: Asc. Prof. Dr. of Medical Microbiology

Nagihan Ozlu: Specialist Dr. in Medical Microbiology

Isin Akyar: Specialist Dr. in Medical Microbiology

Handan Ankarali: Asc. Prof. Dr. of Biostatistics

## Pre-publication history

The pre-publication history for this paper can be accessed here:

http://www.biomedcentral.com/1471-2334/9/30/prepub
